# Robotic Versus Laparoscopic Versus Open Surgery for Rectal Cancer

**DOI:** 10.3390/jcm14196743

**Published:** 2025-09-24

**Authors:** Zsolt Madarasz, Michael Leitz, Miljana Vladimirov, Kira Baginski, Annika Hoyer, Jens Hoeppner, Fabian Nimczewski, Krzysztof Nowakowski

**Affiliations:** 1 Department of Surgery, Medical School and University Medical Center OWL, Bielefeld University, Campus Lippe, 32756 Detmold, Germany; michael.leitz@klinikum-lippe.de (M.L.); miljana.vladimirov@gmail.com (M.V.); jens.hoeppner@klinikum-lippe.de (J.H.); fabian.nimczewski@klinikum-lippe.de (F.N.); krzysztof.nowakowski@klinikum-lippe.de (K.N.); 2 Biostatistics and Medical Biometry, Medical School OWL, Bielefeld University, 33615 Bielefeld, Germany; kira.baginski@uni-bielefeld.de (K.B.); annika.hoyer@uni-bielefeld.de (A.H.)

**Keywords:** rectal cancer, total mesorectal excision, robotic surgery, laparoscopic surgery, open surgery

## Abstract

**Background**: Surgery for rectal cancer has evolved, with the adoption of minimally invasive and robotic techniques. This study evaluates whether robotic surgery for rectal cancer produces an improvement in perioperative and oncological outcomes compared with open and laparoscopic surgery in a retrospective analysis. **Methods**: A single-center retrospective study included 212 patients with histologically confirmed rectal cancer treated between 2012 and 2021. The patients were grouped by surgical approach: robotic (RR, *n* = 62), laparoscopic (LR, *n* = 68), and open resection (OR, *n* = 82). The primary endpoints were total mesorectal excision (TME) quality, operative time, and hospital stay. The secondary endpoints included the lymph node yield, the conversion rate, and the 5-year survival outcomes (OS and DFS), analyzed via Kaplan–Meier curves and proportional hazards models. **Results**: The TME quality was high across the groups (RR: 91.9%, LR: 86.8%, OR: 95.1%). The median operative time was the longest in the RR group (304 min vs. 221–222 min in LR/OR). Robotic surgery resulted in shorter median hospital stays (10 (RR) vs. 14 (LR) vs. 14 (OR) days) and a lower conversion rate (3.2% vs. 14.7% in LR). The lymph node yield was highest in the LR group (27.9), followed by the RR (25.5) and OR (23.0) groups. Postoperative pneumonia was most common in the OR group (12.2%), and bladder dysfunction occurred only in the OR group (4.9%). The five-year OS and DFS did not differ notably between the groups. **Conclusions**: Robotic surgery offers advantages in short-term outcomes, including fewer complications, shorter hospitalization, and lower conversion rates, despite longer operative times. Its oncological efficacy is equal compared to those of laparoscopic and open surgery.

## 1. Introduction

Rectal cancer poses a significant and persistent challenge to public health, ranking among the most common malignancies of the gastrointestinal tract [1]. Traditionally, open rectal resection is associated with considerable morbidity, including prolonged hospitalization, higher infection rates, and significant physical trauma [2]. The desire to mitigate these drawbacks has driven the increasing adoption of minimally invasive techniques, with the goals of enhancing patient recovery and minimizing surgical complications [3]. While minimally invasive techniques, particularly laparoscopic and robotic surgery, have demonstrated non-inferiority to open surgery in terms of long-term survival [4], there is an ongoing debate regarding their impact on the quality of total mesorectal excision (TME) and the completeness of oncologic resection. Several studies have highlighted discrepancies in TME quality between the laparoscopic and robotic procedures, which may influence local recurrence and functional outcomes [5,6,7]. This study aims to provide further real-world evidence to help clarify these aspects.

Laparoscopic surgery, introduced in the early 1990s, offered initial advantages, such as reduced postoperative pain, shorter hospital stays, and a quicker return to daily activities [3]. However, its limitations, including restricted instrument mobility and two-dimensional visualization, can compromise the outcomes, particularly in complex anatomical situations or with lower rectal tumors [8]. Different trials, like MRC CLASSIC, COLOR II, ACOSOG Z6051, and ALaCaRT, have compared laparoscopic and open surgery, generally supporting the feasibility of laparoscopy, but also raising concerns regarding oncologic adequacy, particularly concerning the circumferential resection margins [9,10,11,12].

To overcome the technical limitations of laparoscopy, robotic-assisted surgery was developed. This approach offers three-dimensional visualization, improved ergonomics, and enhanced instrument dexterity [8]. The ROLARR trial demonstrated that robotic assistance resulted in lower conversion rates to open surgery compared to laparoscopy, although the long-term oncological outcomes were similar [13]. More recently, the REAL trial [14] reported improved short-term outcomes with robotic rectal surgery, including less circumferential margin positivity, fewer complications, and faster gastrointestinal recovery. These findings are further supported by additional studies and meta-analyses, especially in patients with mid and lower rectal tumors [15,16]. Despite these advancements, the adoption of robotic surgery is still debated due to the higher costs compared to those of laparoscopic approaches and the need for specialized training [17].

The optimal surgical approach for rectal cancer remains a subject of ongoing debate, particularly concerning the balance between short-term recovery and long-term oncologic safety. This retrospective study aims to compare open, laparoscopic, and robotic rectal resections within a real-world clinical setting. The primary objective of the present study is to provide a comprehensive assessment of the perioperative outcomes, the complication rates, and the oncological results across these three techniques, offering insights into their relative effectiveness and potential benefits.

## 2. Materials and Methods

This retrospective study was conducted at the Department of Surgery, University Hospital OWL Campus Lippe at Bielefeld University. We included patients who underwent rectal resection with partial or total mesorectal excision (PME/TME) with primary anastomosis for histologically confirmed rectal cancer between 1 January 2012 and 31 December 2021. Ethical approval was obtained from the Ethics Committee of the Westfalen-Lippe Medical Association and the University of Münster (Ref. No. 2022-554-f-S).

Between 2012 and 2021, a total of 429 rectal cancer resections were performed. During this period, the surgical practice evolved at the conducting institution; open surgery was the standard approach from 2012 to 2015, followed by increasing adoption of laparoscopic techniques between 2015 and 2018. In 2018, the robotic technique, da Vinci X surgical system (Intuitive Surgical, Sunnyvale, CA, USA), was introduced, leading to a quick transition to robotic rectal resections.

**Figure 1:**
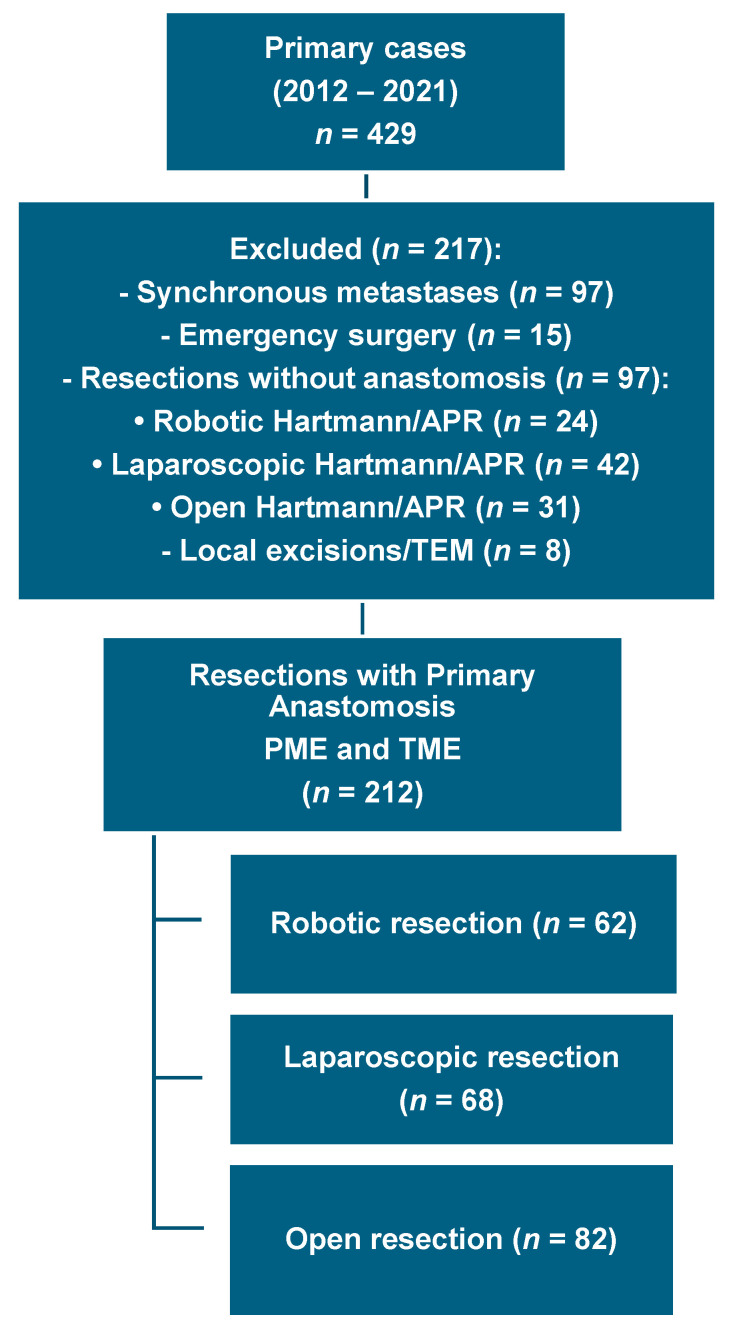
Flow chart of patient selection. APR = abdominoperineal resection; TEM = transanal endoscopic microsurgery; PME = partial mesorectal excision; TME = total mesorectal excision.

The patients were categorized into three groups based on the surgical approach: robotic resection (RR; *n* = 62), laparoscopic resection (LR; *n* = 68), and open resection (OR; *n* = 82). The exclusion criteria were presence of synchronous metastases (hepatic, pulmonary, or peritoneal) and non-restorative procedures, such as Hartmann’s procedure and abdominoperineal resection, and local tumor excision, using transanal endoscopic microsurgery and emergency surgery Figure 1.

All the patients were treated according to the current version of the German S3 guideline for colorectal cancer that was valid at the time of treatment. For reference, the most recent edition is the S3 Guideline Colorectal Cancer, Version 2.1 [18]. Treatment decisions were made in each case by a multidisciplinary tumor board involving surgical, medical, and radiation oncologists, as well as radiologists and pathologists. Neoadjuvant therapy was administered according to the respective version of the German S3 guideline valid at the time of treatment. All patients receiving neoadjuvant treatment underwent long-course chemoradiotherapy consisting of 5-fluorouracil (5-FU) or capecitabine in combination with pelvic radiotherapy (total dose 50.4 Gy). Surgery was typically scheduled from 6 to 10 weeks after completion of chemoradiotherapy. Total neoadjuvant therapy (TNT) was not employed during the study period, as it was only introduced at our institution in 2022.

The data were extracted from a prospectively maintained institutional colorectal cancer database. Comorbidity status was assessed using the American Society of Anesthesiologists (ASA) physical status classification system. This internationally accepted score served as a surrogate marker for global comorbidity burden, as detailed clinical diagnoses (e.g., Charlson index) were not uniformly available in the retrospective dataset. All robotic and laparoscopic procedures were performed by experienced colorectal surgeons. Open resections were partly performed by senior surgical residents under the supervision of consultant surgeons. Bowel preparation and oral antibiotic prophylaxis were administered preoperatively in accordance with institutional standards at the time of treatment. The quality of total mesorectal excision (TME) was assessed according to the Quirke classification. Grade 1 indicates an intact mesorectum with a smooth surface and no defects; Grade 2 refers to moderate irregularities or superficial defects in the mesorectal fascia; and Grade 3 denotes significant defects, tears, or incomplete resection of the mesorectal envelope [19].

Since 2016, intraoperative perfusion assessment using fluorescence angiography with indocyanine green (ICG) has been established as standard practice and was performed in all cases [20].

This study is reported in accordance with the STROBE guidelines for observational studies [21]. Artificial intelligence (AI) tools were used to help with language editing and grammar checking. The authors had full responsibility for the study design, data analysis, and interpretation.

## 3. Statistical Analysis

The patient characteristics were first analyzed descriptively using the mean (standard deviation) or median (Q1; Q3) for continuous variables and proportion (percentage) for categorical variables. Kaplan–Meier curves were used to assess overall survival (OS) and disease-free survival (DFS) across the three surgical methods for rectal cancer resections. For OS, events were defined as deaths occurring within 5 years after surgery. DFS was defined as the time from surgery to the occurrence of either local recurrence, distant metastasis, or death from any cause within the 5-year follow-up period. A proportional hazards model was employed to assess the impact of age, sex, surgical approach, UICC stage, local recurrence, and distant metastasis on OS and DFS. Patients who achieved a pathological complete response (pCR; pathological T0 stage), indicating they were in complete remission, were excluded from the proportional hazard model due to their very low numbers and the absence of events in this subgroup. Hazard ratios (HRs) with 95% Confidence Intervals (CIs) were used as effect measures. A *p*-value of <0.05 was considered statistically significant. All analyses were conducted using R software (version 4.5.0).

## 4. Results

### 4.1. Baseline Characteristics

The baseline characteristics of the study cohort are summarized in Table 1. While the mean age differed slightly across the groups, with the laparoscopic group being younger, the other variables were balanced. These included sex distribution, body mass index, preoperative hemoglobin levels, tumor markers (CEA and CA 19-9), prior abdominal surgery, and neoadjuvant treatment. The open group had a higher proportion of ASA III–IV patients, indicating a greater preoperative risk profile.

### 4.2. Operative Details

The operative characteristics are presented in Table 2. The median operative time was longer in the robotic group (304 min) compared to the laparoscopic (221 min) and open groups (222 min). Conversion to open surgery occurred in 14.7% of the laparoscopic cases and in 3.2% of the robotic cases. The majority of tumors were located in the middle rectum across all the groups. A higher proportion of lower rectal tumors was observed in the robotic group (32.3%) compared to the laparoscopic (19.1%) and open (23.2%) groups. Side-to-end anastomosis was the most commonly performed technique in all the groups, particularly in the robotic cohort (67.7%). Protective stomas were created in more than 80% of patients, with the highest rate in the robotic group (91.9%).

### 4.3. Postoperative Outcomes and Complications

The postoperative outcomes are summarized in Table 3. The robotic group demonstrated the most favorable complication profile overall. Bladder dysfunction was observed exclusively in the open group (4.9%), while the incidence risk of postoperative pneumonia was markedly lower in the robotic group (1.6%) compared to the open group (12.2%) and the laparoscopic group (1.5%). The rate of anastomotic leakage was lowest in the robotic group (11.3%) and highest in the open group (18.3%). The reoperation rates were comparable among the groups. Robotic surgery was associated with the shortest postoperative hospital stay (median 10 days), in contrast to both the laparoscopic and open approaches (14 days each). The duration of ICU stay did not differ substantially between the groups. The proportion of patients experiencing severe complications (Clavien–Dindo grade ≥ IIIb) was lower in the laparoscopic group (8.8%), but comparable between the robotic (16.1%) and open (15.9%) groups. The 30-day mortality risk was low across all the groups (≤1.6%).

### 4.4. Pathological Outcomes

The pathological findings are summarized in Table 4. The quality of total mesorectal excision (TME), assessed by the MERCURY grading system, was high across all the groups, with the highest likelihood of Grade 1 specimens observed in the open group (95.1%), followed by the robotic (91.9%) and laparoscopic (86.8%) groups. R0 resection was achieved in over 98% of cases in all three groups. The mean number of harvested lymph nodes was highest in the laparoscopic group (27.9), followed by robotic (25.5) and open surgery (23.0). Tumor grading and resection margin status were comparable among the groups. Regarding tumor staging, the laparoscopic group showed a higher proportion of UICC stage III tumors (39.7%), while the robotic group had the lowest proportion of advanced-stage disease (22.6%). A pathological complete response (pCR) was observed in 5.7% of the entire cohort.

### 4.5. Survival Analysis

**Figure 2:**
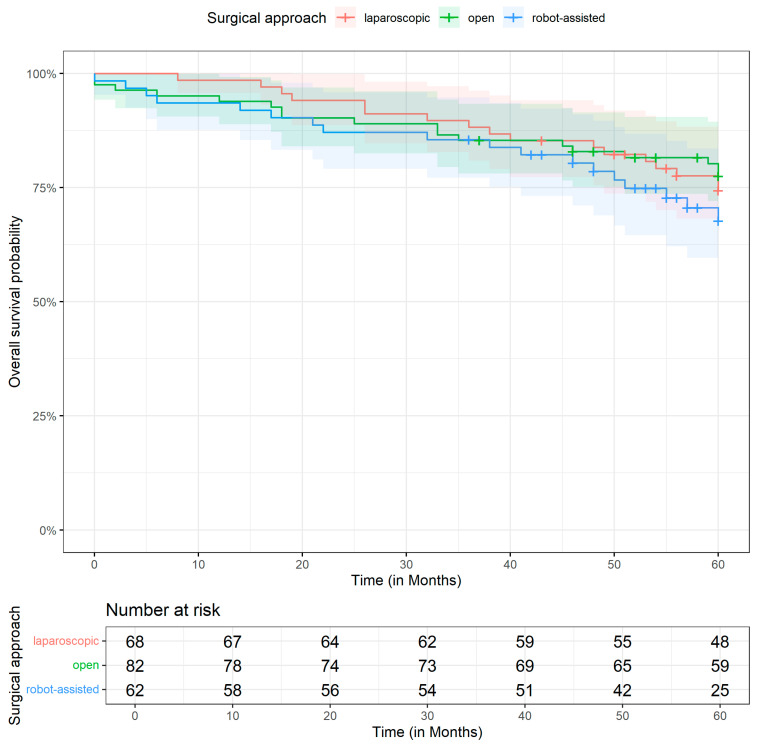
Kaplan–Meier curve for overall survival by operative technique.

**Figure 3:**
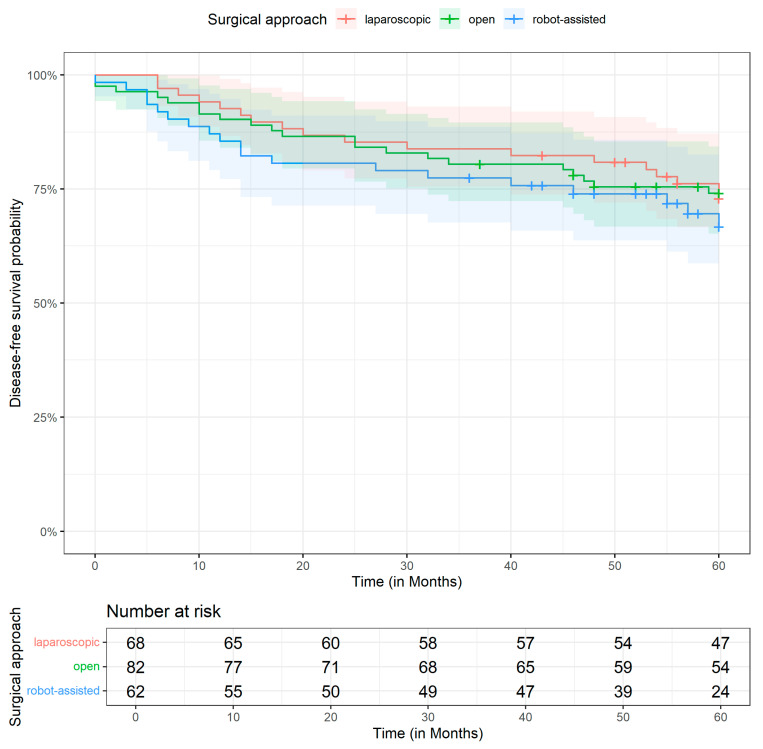
Kaplan–Meier curve for disease-free survival by operative technique.

Figure 2 and Figure 3 display the Kaplan–Meier curves for OS and DFS differentiated by the surgical method. The median follow-up time was 60.0 months (Q1–Q3: 50.0–60.0 months). All the patients were followed at regular intervals in accordance with the national guidelines and had adequate follow-up information for survival analysis. The 5-year OS probabilities are 74.4% [95% CI: 64.6; 85.7] in the laparoscopic group, 77.5% [68.9; 87.3] in the open group, and 67.7% [56.2; 81.7] in the robotic group. Similarly, the 5-year DFS probabilities are 72.9% [62.9; 84.5] in the laparoscopic group, 74.1% [65.2; 84.3] in the open group, and 66.7% [55.2; 80.7] in the robotic group.

Table 5 and Table 6 show the results from the proportional hazards models for the OS and the DFS. There is no evidence for an association of either the type of surgical approach, UICC stage, local recurrence, age, or sex with OS or DFS. Distant metastasis is the only statistically significant predictor. The presence of distant metastasis demonstrated a statistically significant association with OS and DFS when compared to the absence of distant metastasis, with hazard ratios of 6.55 ([3.26, 13.2]; *p* < 0.001) and 10.5 ([5.22, 21.0]; *p* < 0.001), respectively. These findings indicate that patients with distant metastasis face a hazard of death and disease recurrence that is over six or ten times greater compared to those who do not have distant metastasis.

## 5. Discussion

This single-center retrospective study evaluated the perioperative and long-term oncological outcomes of open, laparoscopic, and robotic rectal resections performed over a 10-year period at a tertiary referral center. While all three surgical modalities demonstrated comparable oncological efficacy with respect to the R0 resection rates, TME quality, and long-term survival, significant differences were observed in the perioperative outcomes. These findings are consistent with the existing literature and contribute real-world data to the growing body of evidence surrounding minimally invasive and robotic techniques [13,14].

The proportion of patients receiving neoadjuvant therapy (48.6%) reflects clinical decision making in line with the German S3 guideline valid at the time of diagnosis. All the patients were staged according to the guideline criteria, and treatment recommendations were formulated in an interdisciplinary tumor board. In elderly or medically frail patients, long-course chemoradiotherapy was used more selectively based on individual fitness, comorbidity profile, and expected tolerability. This personalized approach is consistent with real-world clinical practice and likely contributed to the observed rate of upfront resections. Patients who received neoadjuvant therapy typically presented with more advanced or low-lying rectal tumors, which may increase the technical complexity of surgical resection due to fibrosis and altered tissue planes. In this context, robotic surgery may offer advantages through improved visualization and instrument control. However, due to the retrospective, non-randomized nature of the study and the lack of standardized data on tumor response to neoadjuvant therapy, its direct influence on surgical approach selection cannot be definitively assessed.

The robotic approach was associated with the longest operative times, a known limitation widely reported in the literature [16,20]. However, this was offset by favorable short-term outcomes, including substantially shorter postoperative hospital stays and a notably lower conversion rate to open surgery. These advantages align with the findings from the ROLARR and REAL trials. The lower conversion rate with robotic surgery is particularly noteworthy, as it can reduce the morbidity associated with unplanned open conversion [13,14].

Robotic surgery also showed a reduction in postoperative complications, particularly pneumonia and urinary dysfunction, supporting the hypothesis that enhanced pelvic visualization and precise nerve-sparing dissection may translate into improved functional recovery. The preservation of urinary function is a significant benefit, contributing to improved quality of life for patients [22,23].

A total of 85.8% of patients in our cohort received a diverting stoma at the time of rectal resection. This relatively high rate can be attributed to the predominance of mid- and low rectal tumors (70%), as well as the use of neoadjuvant chemoradiotherapy in nearly half of the patients (48.6%). Both these factors are known to increase the risk of anastomotic leakage, and are therefore considered standard indications for protective stoma creation. While diverting ileostomies can reduce the clinical severity of anastomotic dehiscence [24], they also carry their own risks, such as small bowel obstruction and wound-related complications [25,26]. The decision to create a protective stoma must therefore balance these benefits and drawbacks based on individual patient risk profiles.

The oncological surrogate markers, such as TME quality and R0 resection margins, were uniformly high across all the groups, reinforcing the technical feasibility of both minimally invasive approaches in oncological rectal surgery [20]. The slightly higher lymph node yield observed in the laparoscopic group may reflect heterogeneity in pathological work-up or case selection rather than technical superiority. Similar trends have been observed in previous retrospective series and meta-analyses [15,16].

Our survival analysis confirmed that distant metastasis was the most important predictor of both overall and disease-free survival. There was no evidence that the surgical approach, age, the UICC stage, local recurrence, or sex was associated with long-term oncological outcomes, consistent with the findings of multiple randomized trials and registry-based studies [13,23,27].

While the long-term outcomes were similar among the groups, robotic surgery demonstrated clear advantages in short-term parameters. The notably low conversion rate (3.2%) and reduced complication burden may be particularly relevant in male patients, obese individuals, and those with low-lying rectal tumors, where laparoscopic surgery is technically more demanding [23,28]. Furthermore, robotic rectal surgery facilitates precise dissection in narrow pelvises and may reduce the risk of autonomic nerve injury [16,29]. Meta-analyses have consistently shown that robotic surgery offers lower conversion and reoperation rates, as well as improved circumferential resection margin (CRM) clearance in selected patients [15].

However, it is important to balance these advantages against the higher costs and longer operative time, particularly in low-volume settings. The cost-effectiveness of robotic surgery remains a topic of ongoing debate [17,30].

This study has several limitations inherent to its retrospective and single-center design. Although the baseline characteristics appeared comparable across the groups, the absence of randomization introduces the potential for selection bias and residual confounding. The surgical approach was determined by institutional practice, surgeon preference, and resource availability, all of which evolved over a 10-year inclusion period. Importantly, both laparoscopic and robotic techniques were introduced sequentially during the study period. As a result, the observed differences in operative time and short-term outcomes may partly reflect learning curve effects and temporal confounding rather than the surgical modality itself. These factors, in combination with unmeasured clinical variables, may have influenced group allocation and treatment outcomes independently of tumor stage or patient characteristics.

Furthermore, this study did not assess functional outcomes, quality of life, or cost-effectiveness, parameters that are increasingly relevant in evaluating surgical strategies for rectal cancer. These aspects should be addressed in future prospective, preferably multicenter, studies with standardized data collection and a long-term follow-up.

Another relevant limitation is the absence of a transanal total mesorectal excision (TaTME) group. Although TaTME has gained importance in recent years, particularly for tumors of the lower rectum and in anatomically complex cases, this technique was not implemented at our institution during the study period. In 2018, the robotic platform was introduced as the next step in the development of minimally invasive rectal surgery. Since then, three colorectal surgeons were formally trained in robotic techniques, which became the primary advanced approach at our center. Consequently, TaTME was not established as a clinical standard, and no patients underwent this technique in the current cohort. This limitation should be taken into account when interpreting the generalizability of our results in the context of modern rectal cancer surgery.

Nonetheless, this study offers valuable insights from a high-volume European center with consistent surgical protocols and long-term follow-ups. As robotic systems become more accessible and training pathways mature, further high-quality prospective studies and comprehensive cost-benefit analyses are needed to define the optimal role of robotic surgery in rectal cancer treatment [13,14,27].

## 6. Conclusions

In this retrospective cohort study, robotic rectal surgery achieved oncological outcomes similar to those of laparoscopic and open approaches. Robotic surgery offered advantages in terms of lower conversion rates, fewer postoperative complications, and shorter hospital stays. These findings suggest that robotic surgery is a safe and effective technique for rectal cancer resection, particularly in settings where these perioperative benefits are highly valued.

## Figures and Tables

**Table 1:** Baseline characteristics.

	Laparoscopic (*n* = 68)	Open (*n* = 82)	Robotic (*n* = 62)	Overall (*n* = 212)
**Age (in years)**				
Mean (SD)	65.1 (12.0)	69.3 (9.70)	68.1 (12.2)	67.6 (11.3)
**Sex**				
Females	27 (39.7%)	31 (37.8%)	17 (27.4%)	75 (35.4%)
Males	41 (60.3%)	51 (62.2%)	45 (72.6%)	137 (64.6%)
**BMI (kg/m^2^)**				
Mean (SD)	26.1 (4.93)	25.6 (3.48)	26.2 (4.14)	25.9 (4.17)
**ASA score**				
1	6 (8.8%)	1 (1.2%)	16 (25.8%)	23 (10.8%)
2	33 (48.5%)	41 (50.0%)	20 (32.3%)	94 (44.3%)
3	22 (32.4%)	33 (40.2%)	20 (32.3%)	75 (35.4%)
4	7 (10.3%)	7 (8.5%)	6 (9.7%)	20 (9.4%)
**Comorbidities**				
Arterial hypertension	36 (52.9%)	50 (61.0%)	42 (67.7%)	128 (60.4%)
Type II Diabetes mellitus	7 (10.3%)	19 (23.2%)	6 (9.7%)	32 (15.1%)
Coronary artery disease (CAD)	15 (22.1%)	16 (19.5%)	10 (16.1%)	41 (19.3%)
Atrial fibrillation (AF)	6 (8.8%)	12 (14.6%)	10 (16.1%)	28 (13.2%)
Smoking	19 (27.9%)	16 (19.5%)	14 (22.6%)	49 (23.1%)
**Preoperative Hemoglobin (g/dL)**				
Mean (SD)	13.3 (2.04)	13.2 (1.98)	13.1 (1.92)	13.2 (1.97)
**Preoperative CEA (ng/mL)**				
Mean (SD)	5.97 (11.7)	4.47 (8.22)	5.12 (7.12)	5.14 (9.20)
**Preoperative CA 19-9**				
Mean (SD)	13.5 (15.6)	14.4 (31.2)	12.4 (12.9)	13.5 (22.3)
**Previous abdominal surgery**	15 (22.1%)	18 (22.0%)	16 (25.8%)	49 (23.1%)
**Neoadjuvant treatment**	29 (42.6%)	44 (53.7%)	30 (48.4%)	103 (48.6%)

**Table 2:** Operative details.

	Laparoscopic (*n* = 68)	Open (*n* = 82)	Robotic (*n* = 62)	Overall (*n* = 212)
**High of Tumor from anal verge (cm)**				
Mean (SD)	9.96 (3.74)	9.24 (3.18)	8.53 (3.30)	9.26 (3.43)
**Tumor location**				
Lower rectum	13 (19.1%)	19 (23.2%)	20 (32.3%)	52 (24.5%)
Middle rectum	27 (39.7%)	44 (53.7%)	26 (41.9%)	97 (45.8%)
Upper rectum	28 (41.2%)	19 (23.2%)	16 (25.8%)	63 (29.7%)
**Anastomosis technique**				
End-to-End	24 (35.3%)	21 (25.6%)	16 (25.8%)	61 (28.8%)
Side-to-End	31 (45.6%)	48 (58.5%)	42 (67.7%)	121 (57.1%)
ColonJPouch Stapler	7 (10.3%)	11 (13.4%)	2 (3.2%)	20 (9.4%)
ColonJPouch Hansewn	3 (4.4%)	1 (1.2%)	0 (0%)	4 (1.9%)
Colo-anal End-to-End Handsewn	3 (4.4%)	1 (1.2%)	2 (3.2%)	6 (2.8%)
**Operative time (min)**				
Median [Q1, Q3]	221 [181, 264]	222 [191, 247]	304 [260, 361]	242 [195, 295]
**Conversion rate to open surgery**	10 (14.7%)	-	2 (3.2%)	12 (5.7%)
**Protective stoma**	55 (80.9%)	70 (85.4%)	57 (91.9%)	182 (85.8%)

**Table 3:** Postoperative outcomes and complications.

	Laparoscopic (*n* = 68)	Open (*n* = 82)	Robotic (*n* = 62)	Overall (*n* = 212)
**Bowel atony**	9 (13.2%)	15 (18.3%)	5 (8.1%)	29 (13.7%)
**Wound infection**	1 (1.5%)	8 (9.8%)	4 (6.5%)	13 (6.1%)
**Pneumonia**	1 (1.5%)	10 (12.2%)	1 (1.6%)	12 (5.7%)
**Urinary tract infection**	2 (2.9%)	0 (0%)	0 (0%)	2 (0.9%)
**Bladder emptying disorder**	0 (0%)	4 (4.9%)	0 (0%)	4 (1.9%)
**Renal insufficiency**	0 (0%)	2 (2.4%)	2 (3.2%)	4 (1.9%)
**Anastomotic leak**	11 (16.2%)	15 (18.3%)	7 (11.3%)	33 (15.6%)
**Reoperation**	6 (8.8%)	12 (14.6%)	9 (14.5%)	27 (12.7%)
**Clavien–Dindo ≥ II**	17 (25.0%)	36 (43.9%)	13 (21.0%)	66 (31.1%)
**Clavien–Dindo ≥ IIIb**	6 (8.8%)	13 (15.9%)	10 (16.1%)	29 (13.7%)
**Postoperative blood transfusion**	2 (2.9%)	18 (22.0%)	2 (3.2%)	22 (10.4%)
**30-day mortality**	0 (0%)	1 (1.2%)	1 (1.6%)	2 (0.9%)
**Postoperative intensive care unit (ICU) stay (days)**				
Median [Q1, Q3]	1.00 [1.00, 2.00]	2.00 [1.00, 3.00]	1.00 [1.00, 2.75]	1.00 [1.00, 3.00]
**Postoperative length of hospital stays (days)**				
Median [Q1, Q3]	14.0 [9.00, 18.0]	14.0 [12.0, 19.8]	10.0 [8.00, 16.8]	13.5 [10.0, 18.3]
**Local recurrence**	3 (4.4%)	5 (6.1%)	4 (6.5%)	12 (5.7%)
**Distant metastasis**	9 (13.2%)	11 (13.4%)	8 (12.9%)	28 (13.2%)

**Table 4:** Pathological outcomes.

	Laparoscopic (*n* = 68)	Open (*n* = 82)	Robotic (*n* = 62)	Overall (*n* = 212)
**Pathological T stage**				
T0, pCR	5 (7.4%)	3 (3.7%)	4 (6.5%)	12 (5.7%)
T1	12 (17.6%)	10 (12.2%)	9 (14.5%)	31 (14.6%)
T2	20 (29.4%)	25 (30.5%)	22 (35.5%)	67 (31.6%)
T3	26 (38.2%)	41 (50.0%)	24 (38.7%)	91 (42.9%)
T4	5 (7.4%)	3 (3.7%)	3 (4.8%)	11 (5.2%)
**Pathological N stage**				
N0	47 (69.1%)	62 (75.6%)	50 (80.6%)	159 (75.0%)
N1	20 (29.4%)	14 (17.1%)	10 (16.1%)	44 (20.8%)
N2	1 (1.5%)	6 (7.3%)	2 (3.2%)	9 (4.2%)
**Number of lymph nodes harvested**				
Mean (SD)	27.9 (13.3)	23.0 (11.1)	25.5 (13.3)	25.3 (12.6)
**Tumor Grading**				
G1	6 (8.8%)	4 (4.9%)	5 (8.1%)	15 (7.1%)
G2	57 (83.8%)	70 (85.4%)	52 (83.9%)	179 (84.4%)
G3	5 (7.4%)	8 (9.8%)	5 (8.1%)	18 (8.5%)
**Resection margin**				
R0	67 (98.5%)	81 (98.8%)	62 (100%)	210 (99.1%)
R1	1 (1.5%)	1 (1.2%)	0 (0%)	2 (0.9%)
**Quality of TME (Mercury)**				
Grade 1	59 (86.8%)	78 (95.1%)	57 (91.9%)	194 (91.5%)
Grade 2	4 (5.9%)	3 (3.7%)	4 (6.5%)	11 (5.2%)
Grade 3	5 (7.4%)	1 (1.2%)	1 (1.6%)	7 (3.3%)
**UICC Stage**				
Stage 0	5 (7.4%)	2 (2.4%)	4 (6.5%)	11 (5.2%)
Stage I	28 (41.2%)	26 (31.7%)	25 (40.3%)	79 (37.3%)
Stage II	8 (11.8%)	25 (30.5%)	19 (30.6%)	52 (24.5%)
Stage III	27 (39.7%)	29 (35.4%)	14 (22.6%)	70 (33.0%)

**Table 5:** Proportional hazards model for overall survival adjusting for age, sex, surgical approach, UICC stage, local recurrence, and distant metastasis.

Characteristic	HR ^1^	95% CI ^1^	*p*-Value
**Age**	1.02	[0.99, 1.04]	0.280
**Sex**			
Females vs. Males	1.02	[0.57, 1.83]	0.941
**Surgical approach**			
Robotic vs. Open	1.77	[0.89, 3.49]	0.102
Laparoscopic vs. Open	1.58	[0.78, 3.22]	0.204
**UICC Stage**			
Stage II vs. Stage I	1.19	[0.55, 2.57]	0.661
Stage III vs. Stage I	1.26	[0.62, 2.58]	0.527
**Local recurrence**			
Yes vs. No	1.63	[0.71, 3.72]	0.250
**Distant metastasis**			
Yes vs. No	6.55	[3.26, 13.2]	<0.001

^1^ Abbreviations: CI = Confidence Interval; HR = hazard ratio.

**Table 6:** Proportional hazards model for disease-free survival adjusted for age, sex, surgical approach, UICC stage, local recurrence, and distant metastasis.

Characteristic	HR ^1^	95% CI ^1^	*p*-Value
**Age**	1.01	[0.99, 1.04]	0.333
**Sex**			
Females vs. Males	0.87	[0.50, 1.51]	0.616
**Surgical approach**			
Robotic vs. Open	1.87	[0.96, 3.63]	0.065
Laparoscopic vs. Open	1.53	[0.77, 3.01]	0.222
**UICC Stage**			
Stage II vs. Stage I	1.27	[0.61, 2.66]	0.521
Stage III vs. Stage I	1.35	[0.68, 2.68]	0.397
**Local recurrence**			
Yes vs. No	1.81	[0.84, 3.89]	0.129
**Distant metastasis**			
Yes vs. No	10.5	[5.22, 21.0]	<0.001

^1^ Abbreviations: CI = Confidence Interval; HR = hazard ratio.

## Data Availability

The data supporting the results can be made available on reasonable request from the corresponding author.

## Abbreviations

The following abbreviations are used in this manuscript:

APR	Abdominoperineal resection
TEM	Transanal endoscopic microsurgery
PME	Partial mesorectal excision
TME	Total mesorectal excision
CEA	Carcinoembryonic antigen
CA19-9	Carbohydrate antigen 19-9
OS	Overall survival
DFS	Disease-free survival
ICG	Indocyanine green
UICC	Union for international cancer control
